# Spatial Rabi oscillations between Majorana bound states and quantum dots

**DOI:** 10.3762/bjnano.9.143

**Published:** 2018-05-22

**Authors:** Jun-Hui Zheng, Dao-Xin Yao, Zhi Wang

**Affiliations:** 1 School of Physics, Sun Yat-sen University, Guangzhou 510275, China

**Keywords:** Andreev bound state, Majorana bound state, nonlocal quantum correlation, quantum dot, spatial Rabi oscillation

## Abstract

**Background:** A Majorana bound state is a superconducting quasiparticle that is the superposition of particle and hole with equal amplitude. We propose a verification of this amplitude equality by analyzing the spatial Rabi oscillations of the quantum states of a quantum dot that is tunneling-coupled to the Majorana bound states.

**Results:** We find two resonant Rabi driving energies that correspond to the energy splitting due to the coupling of two spatially separated Majorana bound states. The resulting Rabi oscillating frequencies from these two different resonant driving energies are identical for the Majorana bound states, while different for ordinary Andreev bound states. We further study a double-quantum-dot setup and find a nonlocal quantum correlation between them that is mediated by two Majorana bound states. This nonlocal correlation has the signature of additional resonant driving energies.

**Conclusion:** Our method can be used to distinguish between Majorana bound states and Andreev bound states. It also gives a precise measurement of the energy splitting between two Majorana bound states.

## Introduction

Majorana bound states are exotic non-Abelian quasiparticles in topological superconductors [1–26]. The study of Majorana bound states has attracted tremendous interest recently because they constitute topological parity qubits. These qubits are defined by the degenerate ground states of topological superconductors, and therefore are protected by the superconducting energy gap [4,15]. They have a long coherence time and are resistant to local decoherence sources [15,2,18–19]. Most importantly, the topological qubits can be topologically manipulated by braiding the Majorana bound states [4,15,17]. These topological braiding operations set the foundation for topological quantum computation [15,4], despite the fact that they are insufficient to construct universal quantum gates for the topological qubit [17,19–20,15].

A unique feature of the Majorana bound states is the self-conjugateness. In the language of second quantization, a self-conjugate quasiparticle means that the superposition of the electron creation operators and electron annihilation operators are equal [2,15–16]. This equality is the essential difference between the Majorana bound states and the ordinary Andreev bound states. Another unique feature of Majorana bound states is the exponential protection [18,17,19], which states that the splitting energy between two Majorana bound states exponentially decays as the distance between them increases. The experimental verification of these two properties helps the identification of Majorana bound states in real systems.

Majorana bound states have been theoretically proposed in several systems [9,6,5,20,13], while the experiments concentrate on semiconductors with spin–orbit coupling and the superconducting gap that is induced by the superconducting proximity effect [21,24–25,19]. One promising candidate is the hybrid system of a spin–orbit-coupling nanowire and a conventional superconductor. Robust zero-bias conductance peak was first reported in this system, which originates from the self-conjugate nature of Majorana bound states and therefore was wildly recognized as a signature. An exotic fractional Josephson effect was also studied in the nanowire Josephson junctions, where novel Shapiro steps and Josephson radiations have been reported. Recently, the Coulomb blockade spectroscopy was exploited on finite-size nanowire segments that form nanowire islands with two Majorana bound states possibly existing at the two ends of the island. The splitting energy between two Majorana bound states is found to be decreasing exponentially when the length of the island increases [24]. This exponential protection of zero-energy Majorana bound states stirs new excitement in pursuing Majorana bound states.

Quantum dot has been proved to be a good probe to study the Majorana bound states [27,3,28–36,7,37–38]. The quantum dots are zero-dimensional systems that have controllable discrete energy levels. The Rabi oscillation, a fundamental quantum phenomenon in two-level quantum systems, may occur between the states of the quantum dot when the quantum dot is periodically modulated. In particular, the spatial Rabi oscillation between two quantum dots has been proven to be useful for single-electron pumping. An attractive idea is to exploit the spatial Rabi oscillation between the quantum dots and the Majorana bound states [29] and to investigate the self-conjugateness and exponential protection of Majorana bound states. In recent experiments, a hybrid structure of a quantum dot and a one-dimensional topological superconductor nanowire has been realized [36]. This system attracts theoretical interest [7,37]. In this context, it is interesting to study the spatial Rabi oscillation between the quantum dot and the topological nanowire.

**Figure 1:**
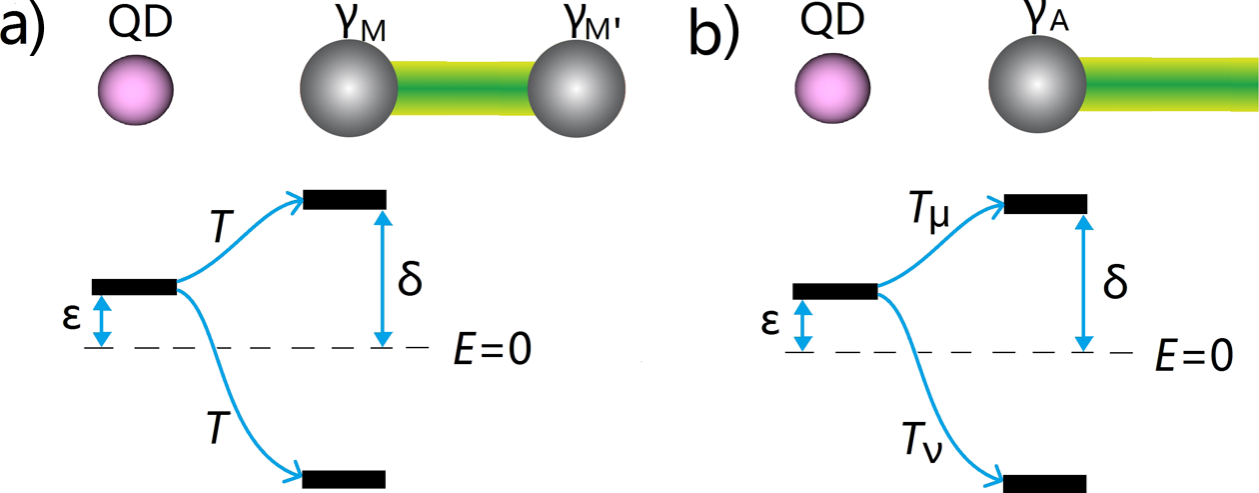
Schematics of a quantum dot tunneling-coupled to a nanowire island with (a) Majorana bound states, and (b) an Andreev bound state. The Andreev bound state has a small excitation energy δ, which is similar to the splitting energy between two Majorana bound states. The effective coupling between the quantum dot and the Andreev bound state has different electron and hole components *T*_μ_ and *T*_ν_, due to the different electron and hole wave functions of the Andreev bound state. In contrary, the effective coupling between the quantum dot and the Majorana bound state has identical electron and hole component *T*, due to the self-conjugateness of the Majorana bound states.

In this work, we study the spatial Rabi oscillations between quantum dots and a Majorana island. This system involves two Majorana bound states that have an exponentially protected small splitting energy. As shown in Figure 1a, one of the Majorana bound states is coupled to the quantum dot with a single electron tunneling through a potential barrier. The barrier is produced by a voltage gate, which is implemented between the quantum dot and the Majorana island. If an ac voltage is applied to the gate, the tunneling strength between the quantum dot and the Majorana bound states will be driven periodically [39]. We show that there are two resonant driving energies that induce coherent spatial Rabi oscillations between the quantum dot and the island. The difference between the two driving energies is proportional to the exponentially protected splitting energy between two Majorana bound states. More importantly, the Rabi frequencies connected to the two different resonant driving energies are identical, which is a result of the self-conjugateness of the Majorana bound states. For comparison, we show the results when the Majorana bound state is replaced by an Andreev bound state as shown in Figure 1b. We find that the two Rabi frequencies at the different resonant driving energies are now different. We also investigate the setup with two quantum dots at each side of the island and calculate the resonant driving energies for spatial Rabi oscillation. We show that the two quantum dots exhibit nonlocal correlations when coupled with Majorana bound states while the two dots have no correlation when coupled with Andreev bound states, since two Majorana bound states can form one single fermionic level while two Andreev bound states are two distinct fermionic levels.

## Results and Discussion

### Model

The hybrid system schematically illustrated in Figure 1 consists of two parts, a quantum dot and a nanowire island, where Majorana or Andreev bound states are present at the ends of the island. Let us first consider the model for the quantum dot. Realistic topological superconducting systems usually involve a large Zeeman field, which in principle should break the spin degeneracy and split the two spin-dependent levels with the Zeeman energy. Therefore, it is reasonable to consider only one-spin direction. Meanwhile, we consider a large Coulomb blockade regime for the quantum dot, which corresponds to a large Coulomb interaction. For this regime, additional electron hopping to the quantum dot requires a large Coulomb energy, which effectively reduces the quantum dot to only one relevant energy level. The Hamiltonian of a minimal model for the quantum dot is [7,3,38,27],

[1]
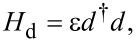


where ε is the excitation energy for the single energy level of the quantum dot and *d*^†^ represents the creation operator on the energy level.

The Majorana island consists of a one-dimensional topological superconductor such as a nanowire–superconductor hybrid structure and a ferromagnetic chain, with zero-energy Majorana bound states at the ends of the system. The wave functions of the two Majorana bound states overlap with each other, inducing an energy splitting that exponentially decays as the length of the island increases. The low-energy physics of the island can be described by an effective Hamiltonian [2–3],

[2]
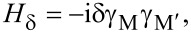


where and γ_M_ and γ_M′_ represent the two Majorana bound states, and δ represents the exponentially protected splitting energy. The quantum dot is coupled to one of the Majorana bound states by electron tunneling through a potential barrier between the dot and the Majorana island. This coupling can be described by a tunneling Hamiltonian,

[3]
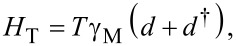


where *T* is the tunneling strength that is taken as a real number for simplicity. Here we consider an oscillating tunneling strength *T* = *T*_0_ + 2*T*_1_cosω*t*, with *T*_0_ being the static tunneling strength, *T*_1_ the oscillating tunneling strength, and ω the oscillating frequency for the tunneling strength. It can be produced by an ac gate voltage controlling the tunneling barrier [39]. When the driving frequency is at resonance, this oscillating tunneling strength can induce a Rabi oscillation on the quantum dot.

We write out the matrix form for the total Hamiltonian *H*_M_ = *H*_d_ + *H*_δ_ + *H*_T_. We first define a new fermionic operator *f*^†^ = (γ_M_ − iγ_M′_)/2, which leads to

[4]
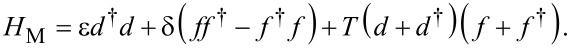


Then we take the four eigenstates of the fermionic operators 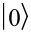, 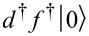, 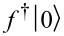, and 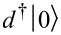 as the basis states of the Hilbert space, and express the Hamiltonian in this basis explicitly,

[5]
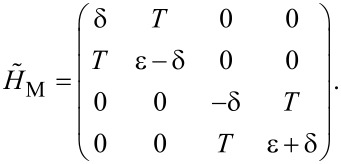


This matrix is block diagonal due to the parity conservation of the total system. We notice that the upper left and the lower right 2 × 2 blocks have the same off-diagonal elements but different diagonal elements.

Now we consider the scenario that the nanowire island has an Andreev bound state at the end instead of Majorana bound states. From the mean-field Bogoliubov–de Gennes approach, the general form for Andreev bound states is the quantum superposition of electron and hole wave function, which in the second quantization form writes as,

[6]
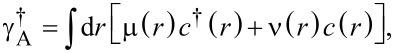


where *c*^†^(*r*) is the creation operator for the electron, μ and ν are the electron and hole wave functions. For the sake simplicity they are real numbers and the factor of 1/2 for describing superconducting quasiparticles is absorbed into μ and ν. We assume the simplest wave function of delta equations since the Andreev bound state is extremely localized at the end of the wire. Then the Andreev operators can be written as 
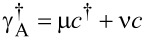
. With this in mind, we can now study the Hamiltonian of the system of a quantum dot and an Andreev bound state. It can be written as *H*_A_ = *H*_d_ + *H*_Ta_ + *H*_a_ where the Hamiltonian for the Andreev bound state, *H*_a_, is

[7]
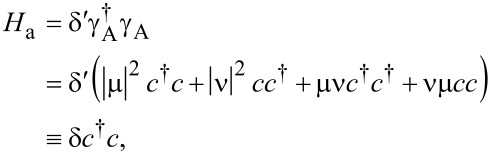


with δ = δ′(|μ|^2^ − |ν|^2^). Due to the particle–hole symmetry of the superconducting system, we can always obtain another Andreev bound state by defining a new operator 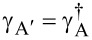. This leads to the excitation energy −δ, which accounts for the negative energy excitations observed in experiments [17,37]. The tunneling Hamiltonian between the Andreev bound state and the quantum dot is,

[8]
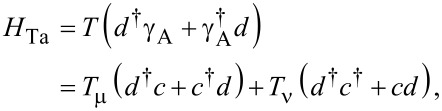


where we define *T*_μ_ = μ*T* and *T*_ν_ = ν*T*. Now we can establish a basis for *H*_A_ with eigenstates 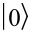, 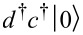, 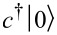 and 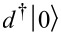, and rewrite in the matrix form,

[9]
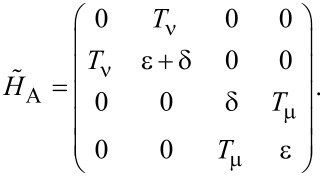


It looks similar to Equation 5 but with one critical difference: The off-diagonal terms in the upper left block and the lower right block are now different because they contain electron and hole wave functions, which are different for Andreev bound states. We note that the Andreev bound state may have equivalent particle and hole components (*u*_↑_ = *v*_↑_) for some spin directions. For this case, the matrices in Equation 5 and Equation 9 are identical if the energy level of the quantum dot is in the same direction. However, the spin direction of the quantum level on the dot can be reversed by inverting the Zeeman field. Then the matrix for the Andreev bound state contains the electron and hole wave functions in the reverse spin direction and must be different.

### Spatial Rabi oscillations

Now we are ready to consider the spacial Rabi oscillations where an electron oscillates between the quantum dot and the bound states. For this purpose we solve the Schrödinger equation 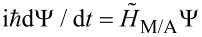. The Hamiltonian is periodic in time, therefore the equation is not exactly solvable. To obtain the Rabi oscillations, we need to study the transition probability from one state to the other under this time periodic Hamiltonian. We take advantage of the Floquet theory, which states that the solution of the Schrödinger equation for any time-periodic Hamiltonian must satisfy Ψ(*t*) = ψ(*t*) *e*^−i^*^Dt^*, with ψ(*t*) = ψ(*t* + (2π/ω) a time-periodic function that has a Fourier transformation 
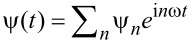
. Let us first consider the scenario of Majorana bound states where we can obtain a series of secular equations by inserting the ansatz solution back into the Schrödinger equation,

[10]
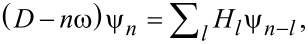


where *l* = 0, ±1, and the Fourier transformed components of the Hamiltonian *H_l_* are

[11]
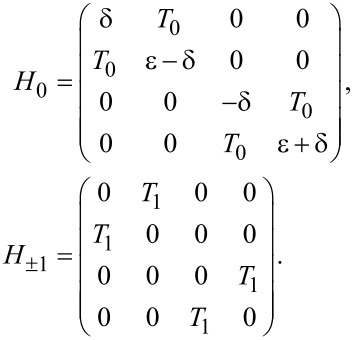


Now the problem of solving a time-dependent Schrödinger equation is transformed to a problem of solving a set of time-independent secular equations [40]. Since ψ*_n_* is a vector with two components, special care is needed when trying to solve the secular equations. They should be rewritten as


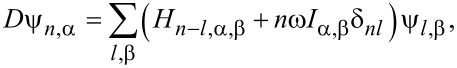


with α,β = 0, 1, 2, 3. Then, the secular equations can be viewed as the eigenproblem for the infinite dimensional Floquet Hamiltonian [40],

[12]
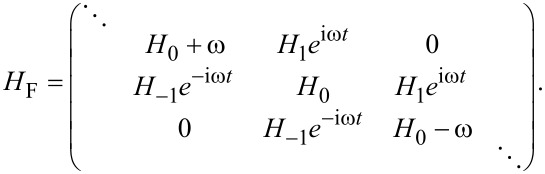


In this Floquet formalism, the transition probability between any two states is written as

[13]
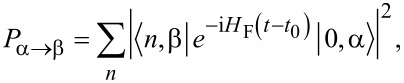


which could be calculated once we solve the eigenproblem for the Floquet Hamiltonian.

In the Floquet Hamiltonian, the off-diagonal elements are between the nearest blocks. For the zero-order perturbation, we first consider the transition between 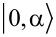 and 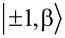. Two cases are studied: α = 0 with β = 1 and α = 2 with β = 3. Then we can extract a 4 × 4 matrix,

[14]
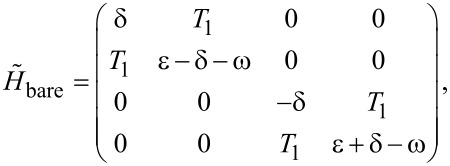


which of course can be divided into two relevant 2 × 2 matrices,

[15]
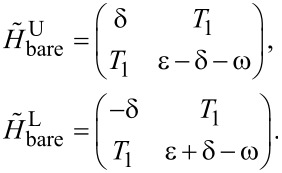


We can also include the second-order perturbation, which slightly alters the diagonal elements of the 2 × 2 matrix,

[16]
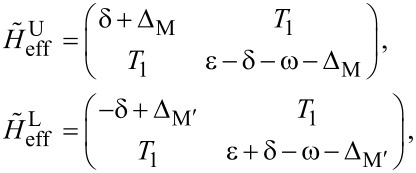


where


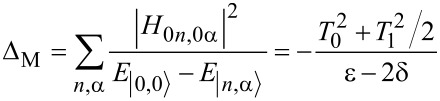


and


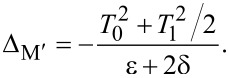


Now the transition probability is clear. Starting from an initial state


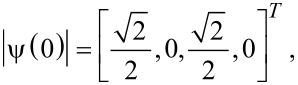


we would have a Rabi oscillation for

[17]
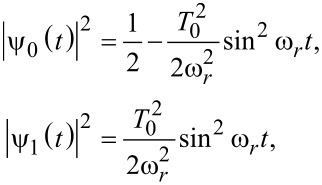


with


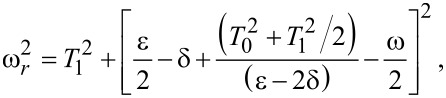


or for

[18]
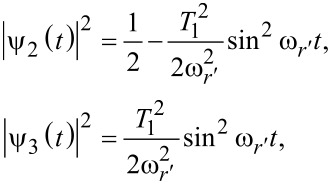


with


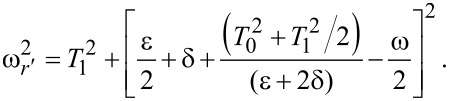


Clearly, there are Rabi oscillations at with two resonant driving energies at


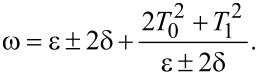


However, for both resonant driving energies, the Rabi frequency is the same ω*_r_* = ω*_r′_* = *T*_1_. This is not a coincidence, but is a result of the self-conjugateness of the Majorana bound states.

Now we consider the scenario of Andreev bound states. With the same Floquet approach, we can obtain the effective Floquet Hamiltonians,

[19]
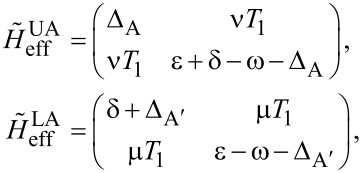


where


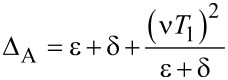


and


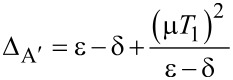


when we set *T*_0_ = 0. Clearly, we also have two resonant driving energies. However, now we have different Rabi frequencies for these two resonant driving energies, ω*_r_* = ν*T*_1_ and −ω*_r_*_′_ = μ*T*_1_, which are given by solving the Floquet Hamiltonians in Equation 19. The difference between the Rabi frequencies comes from different particle and hole wave functions, μ and ν, for the Andreev bound states.

**Figure 2:**
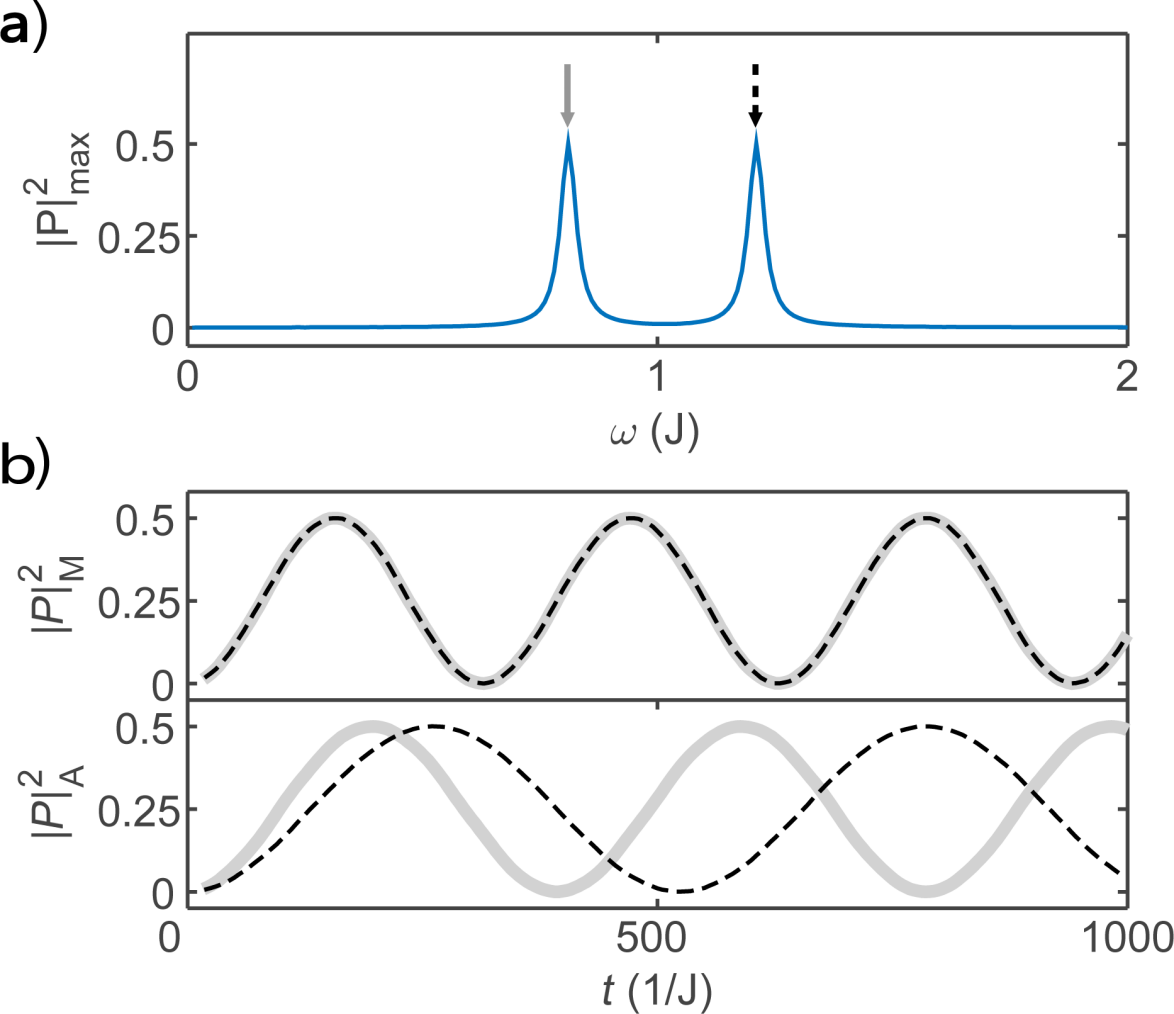
Numerical simulations of the Rabi oscillations. (a) The maximum Rabi oscillation amplitude measured by the occupation probability of the quantum dot, as a function of the driving energy, where the two peaks marks the resonant driving energies. (b) The Rabi oscillation for the two resonant driving energies for the Majorana bound state (upper panel) and the Andreev bound state (lower panel). We see that the Rabi frequencies are identical for the Majorana bound state while they are different for the Andreev bound state. Parameters are taken as ε ≡ *J*, *T*_0_ = 0, δ = 0.1*J*, *T*_1_ = 0.01*J*, δ = 0.2*J*, μ*T*_1_ = 0.008*J* and ν*T*_1_ = 0.006*J* with the initial state 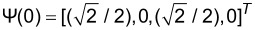.

We present numerical simulations for the hybrid system in Figure 2. First, we show the largest oscillation amplitude on the quantum dot as a function of the driving energy ω in Figure 2a, where the scenario for Majorana bound states and for Andreev bound states present the same result. The two peaks represent the two resonant driving energies. For the Majorana bound state, the energy difference between these two peaks is proportional to the splitting energy between the two Majorana bound states at the ends of the island. Since the measurement of Rabi oscillation is much more accurate than transport measurements, the resonant driving energy provides a precise method to measure the exponential decay of the splitting energy. The Rabi oscillations of the occupation state of the quantum dot for Majorana and Andreev bound states are presented in Figure 2b. We find that the Rabi frequencies of the Majorana bound state are identical as predicted by the analytic results based on the Floquet theory. For comparison, we also present the Rabi frequencies for the Andreev bound state. The Rabi frequencies are different, reflecting the inequality of the electron and hole components for the Andreev bound state.

The results presented in Figure 2 are the central results of our work. We emphasize that these theoretical results can be measured with existing experimental techniques. Our calculation gives the Rabi oscillations of the occupation states of the quantum dot, which can be measured by probing the electron occupation on the quantum dot. The measurement of the electron occupation state of the quantum dot has been achieved with the single-electron transistor [27,41], which is a routine technique in the study of charge qubits based on quantum dots [42].

Finally, we note that our results are based on the minimal models for the quantum dot, the Majorana bound state and the Andreev bound state. It is certainly helpful to consider more sophisticated models for the quantum dot by including the Zeeman energy and Coulomb energy explicitly, and more realistic models for the Majorana bound state and Andreev bound state by exploiting the Bogoliubov–de Gennes Hamiltonian. However, these works are beyond the scope of our current work and belong to our plan of future works.

### Correlation between quantum dots through Majorana islands

**Figure 3:**
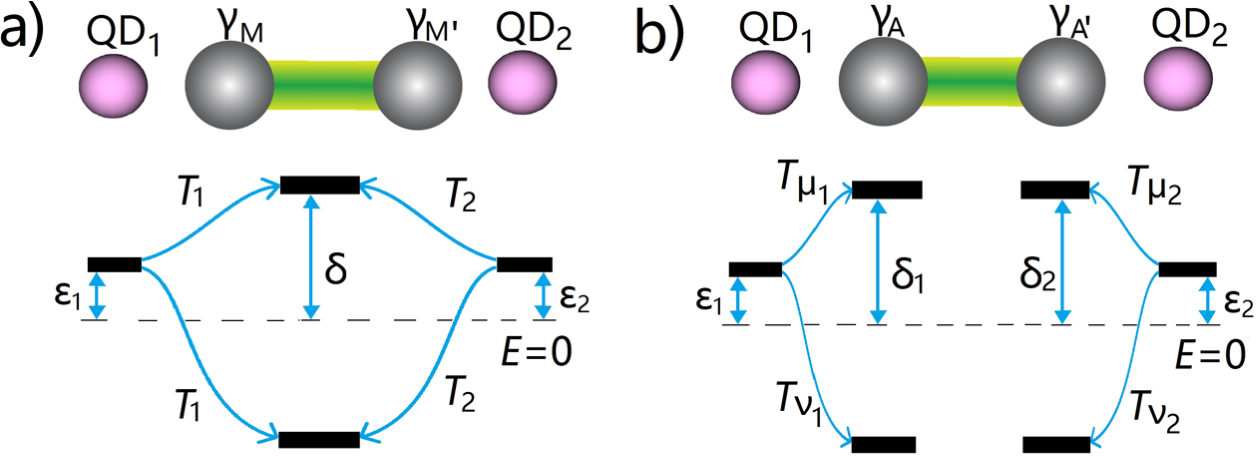
Schematics of two quantum dots coupling with the nanowire island with (a) two Majorana bound states and (b) two Andreev bound states. The two Majorana bound states form a single energy level, while the Andreev bound states form two energy levels.

Now we investigate the setup with two quantum dots on the two sides of a nanowire island, as shown in Figure 3. In this setup, nonlocal entanglement between quantum dots mediated by Majorana bound states has been discussed [28]. It seems logical to consider how this nonlocal entanglement influences the Rabi oscillations. First, each quantum dot certainly has Rabi oscillations with a Majorana bound state or an Andreev bound state at each end. However, we will show a more interesting correlation between quantum dots mediated by two Majorana bound states. This correlation does not occur for the Andreev bound state. Let us first establish the Hamiltonian for the proposed setup. The two quantum dots have the Hamiltonian

[20]
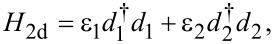


where ε*_1,2_* are the energy for quantum dot levels, and 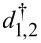 are the creation operators on the quantum dot levels.

The Hamiltonian for the Majorana bound states is the same as in the previous section, which could be described by a fermionic operator *f*^†^ = (γ_M_ − iγ_M′_)/2. The two quantum dots are coupled with the Majorana bound states through the tunneling Hamiltonian

[21]
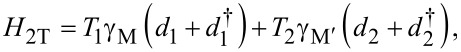


where *T_1,2_* are the tunneling strength between the left and the right pair of quantum dot and Majorana bound state in the form of *T_1,2_* = 2*T*′*_1,2_*cosω*_1,2_t*. We can explicitly write down the total Hamiltonian in the matrix form by defining basis functions 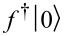, 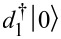, 
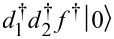
, 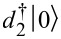, 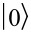, 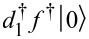, 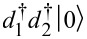 and 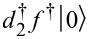, where 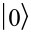 is the vacuum state. We arrive at an 8 × 8 matrix that is block diagonal because the total fermionic parity of the system is conserved. For simplify, we take the even total parity, and get a 4 × 4 matrix,

[22]
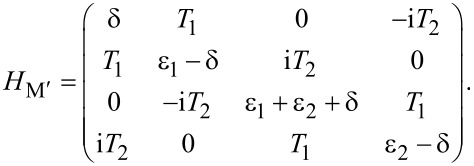


Now let us look at the quantum dots coupling with two Andreev bound states at the end of the nanowire island. Since Andreev bound states are eigenstates of superconductors, there are, in principle, four energy levels in the entire system. The Hamiltonian of the system cam be written as

[23]
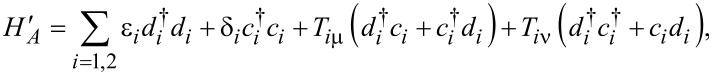


where *i* represents the left/right side of each operator with *T_iμ_* = μ*T_i_*, *T_iν_* = ν*T_i_*. For this case, the system can be divided into left and right segments, which are uncoupled from each other. For simplicity, we take the even parity of both sides, where the basis states are chosen as 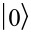, 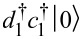, 
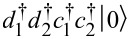
 and 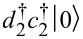. Then the Hamiltonian can be reformed to a four by four matrix:

[24]
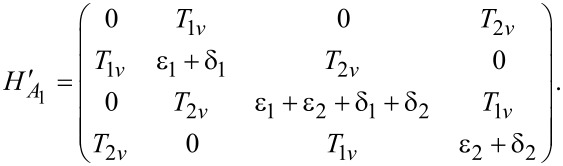


We find that this matrix is very similar to the matrix of quantum dots and Majorana bound states. However, there is the key distinction that the left quantum dot and the right quantum dot should be entirely uncoupled. We note that this matrix is different from the one shown in Equation 22 even if μ = ν, since the two Andreev bound states correspond to two superconducting quasiparticles with a 4 × 4 Hilbert subspace, while the two Majorana bound states gives a single superconducting quasiparticle with a 2 × 2 Hilbert subspace.

**Figure 4:**
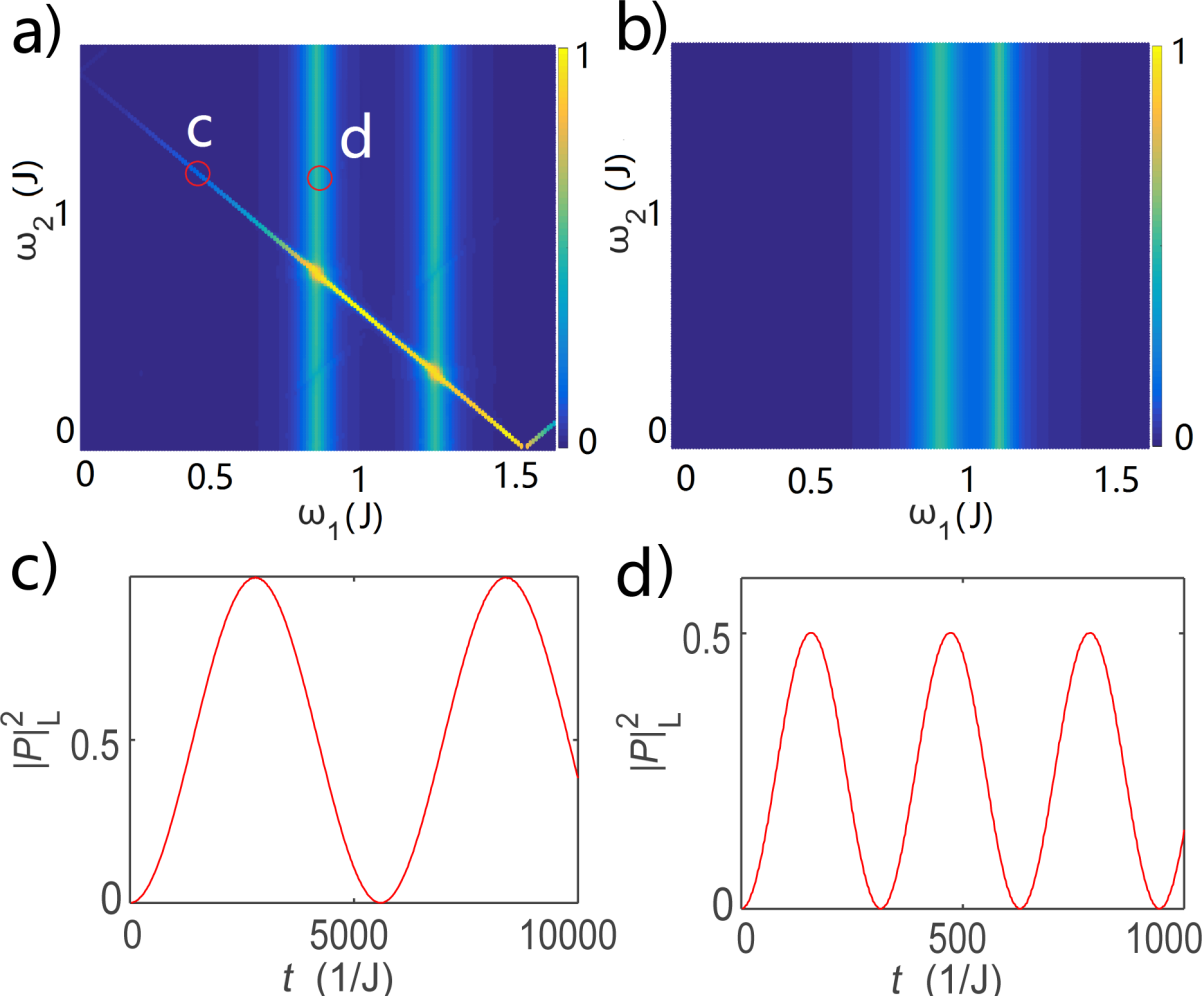
The maximum occupation probability of the left quantum dot for (a) Majorana bound states and (b) Andreev bound states. Panels (c) and (d) give the detailed oscillation as a function of the time at two specific parameters marked as circles on (a).

We numerically simulate the oscillations for the Majorana bound states scenario and illustrate the maximum oscillation amplitude for the occupation state of the left quantum dot in Figure 4a. We find three lines of resonant driving energy. The two vertical lines represent the resonant Rabi oscillation at ω_1_ = ε_1_ ± 2δ, with a typical result shown in Figure 4c. They are identical to the case of the single quantum dot and represents the Rabi oscillation between the left quantum dot and the two Majorana bound states, while leaving the right quantum dot uninvolved. There is one extra line that represents the resonant Rabi driving energy at ω_1_ + ω_2_ = ε_1_ + ε_2_. This resonant energy involves both quantum dots, and therefore would be coming from the nonlocal entanglement of the quantum dots. We present a typical Rabi oscillation in Figure 4d. It is the higher-order oscillations between the states 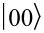 and 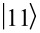, namely a charge oscillation between the left and right quantum dots. This is a nonlocal coherent charge transfer process between the quantum dots mediated by the two Majorana bound states. For comparison, we illustrate the results for quantum dot occupation mediated through Andreev bound states. We find that the Rabi oscillations at ω_1_ ≈ ε_1_ ± δ still exist, however, the higher-order oscillations disappear. This can be explained by the fact that left and the right part of the setup are uncoupled. The extra resonant driving energy for Majorana bound states is a result of the nonlocal quantum dot correlation and can be used as a signature for Majorana bound states.

### Conclusion

We studied the spatial Rabi oscillation between quantum dots and Majorana bound states in a topological superconducting island. We demonstrate that the coupling energy between Majorana bound states can be detected by investigating the resonant driving energy for the Rabi oscillation. We also show that the Rabi oscillating frequency carries the information of the electron and hole components, therefore can be used to differentiate Majorana bound states and Andreev bound states. At the two resonant driving energies, we find identical Rabi frequencies for Majorana bound states and different Rabi frequencies for Andreev bound states. We further study the case of two quantum dots coupled through the island and show that the Majorana bound states are able to create correlated higher-order Rabi oscillations on the quantum dots.
